# Burnout and job satisfaction of psychiatrists in China: a nationwide survey

**DOI:** 10.1186/s12888-021-03568-6

**Published:** 2021-11-24

**Authors:** Hao Yao, Peicheng Wang, Yi-Lang Tang, Yuanli Liu, Tingfang Liu, Huanzhong Liu, Yanhua Chen, Feng Jiang, Jiming Zhu

**Affiliations:** 1 Shanghai Clinical Research Center for Mental Health, Shanghai Key Laboratory of Psychotic Disorders, Shanghai Mental Health Center, Shanghai Jiao Tong University School of Medicine, Shanghai, 200030 China; 2 Vanke School of Public Health, Tsinghua University, Beijing, 100084 China; 3 School of Medicine, Tsinghua University, Beijing, 100084 China; 4 Mental Health Service Line, Atlanta VA Medical Center, Decatur, GA 30033 USA; 5 Addiction Psychiatry Fellowship Program, Department of Psychiatry and Behavioral Sciences, Emory University, Atlanta, GA 30329 USA; 6 School of Health Policy and Management, Chinese Academy of Medical Sciences & Peking Union Medical College, Beijing, 100730 China; 7 Institute for Hospital Management of Tsinghua University, Beijing, 100091 China; 8 Department of Psychiatry, Chaohu Hospital of Anhui Medical University, Hefei, 238000 China; 9 Department of Psychiatry, Anhui Psychiatric Center, Anhui Medical University, Hefei, China; 10 School of International and Public Affairs, Shanghai Jiao Tong University, Shanghai, China

**Keywords:** Burnout, Job satisfaction, Psychiatrists, China

## Abstract

**Background:**

Despite a significant shortage of psychiatrists in China, an ever-increasing number of psychiatrists in China are experiencing burnout and job dissatisfaction and considering leaving their jobs. Yet, to our knowledge, there have been no nationwide studies to date that examined both burnout and job dissatisfaction of psychiatrists in China. Therefore, this study evaluated burnout and job dissatisfaction of psychiatrists in China, and identified relevant characteristics.

**Methods:**

We conducted a nationwide, cross-sectional survey in March 2019. Psychiatrists from all tertiary psychiatric hospitals in China were invited to participate. The Maslach Burnout Inventory-Human Service Survey and the short version of the Minnesota Satisfaction Questionnaire were used to measure burnout and job satisfaction. Data on socio-demographic and occupational characteristics were collected. Multivariate logistic regression was conducted to identify socio-demographic and occupational characteristics associated with burnout and job satisfaction.

**Results:**

In total, 4520 psychiatrists from tertiary psychiatric hospitals in China completed the questionnaire. Overall, 38.4% of respondents met the criteria for burnout and 35.6% were dissatisfied with their jobs. Being male, more years of practice, having no leadership role, and longer working hours per week were significantly associated with burnout and job dissatisfaction. Lower monthly pay was significantly associated with job dissatisfaction but not burnout. Moreover, burnout was significantly associated with job dissatisfaction.

**Conclusions:**

Our data suggest a high rate of burnout and job dissatisfaction among psychiatrists in China. In order to preserve and strengthen the mental health workforce, proactive measures are urgently needed to mitigate burnout and job dissatisfaction among psychiatrists in China.

**Supplementary Information:**

The online version contains supplementary materials available at 10.1186/s12888-021-03568-6.

## Background

With an estimated 173 million people living with mental disorders currently in China [1], China accounts for the largest percentage (17%) of the global mental, neurological, and substance use disorder burden [2]. According to the latest nationwide survey of mental disorders in China, the lifetime prevalence of any mental disorder has increased significantly from 1.3% in 1982 to 16.6% in 2015 [3]. Moreover, since the outbreak of the COVID-19 epidemic, it was suggested that Chinese mental health burden has further escalated [4, 5]. One nationally representative study showed that the prevalence of anxiety or depression or both was as high as 20.4% among the general adult population in China at the peak of the COVID-19 epidemic [6]. Not only do mental disorders have high personal costs for patients and caregivers, but they also have high social and economic costs for the entire society. It was estimated that the year 2013 witnessed a total annual cost of $88.8 billion attributed to mental disorders in China, which was nearly four times that in 2005 [7].

Evidently, China is now faced with a surging demand for mental health service provision among the general population. However, a severe shortage of psychiatrists poses a major challenge to China’s mental health care system. Nowadays, the total number of psychiatrists in China is just above 40,000 [8], indicating that there are less than 3 psychiatrists per 100,000 population. Moreover, most of these psychiatrists are working in the more socioeconomically developed eastern coastal region of China, leaving the treatment gap for mental disorders in China’s western and central regions strikingly huge [8–10]. In order to develop and strengthen the mental health workforce in China, the General Office of China’s State Council issued the *2015–2020 National Mental Health Work Plan* [11] in 2015 which proposed a package of measures to develop and strengthen the mental health workforce in China, including encouraging regions and higher education institutions to provide medical bachelor’s degrees specializing in psychiatry, allowing non-psychiatrist physicians to switch their clinical specialties to psychiatry after receiving training in psychiatry, and improving the level of remuneration for mental health professionals. However, the effectiveness of these measures has been inadequately evaluated and the number of psychiatrists in China is still far less than needed [10]. In addition, most of these efforts till now have been focused on recruiting more psychiatrists, with the retention issues of psychiatrists largely neglected. In fact, it was reported that an ever-increasing amount of psychiatrists in China are experiencing occupational burnout which further leads to job dissatisfaction [12] and intent to leave their jobs [13].

As defined by the World Health Organization [14], burnout is “a syndrome conceptualized as resulting from chronic workplace stress that has not been successfully managed.” It is characterized by three dimensions: emotional exhaustion (EE), depersonalization (DP), and reduced personal accomplishment (PA) [15]. Numerous studies have reported a high rate of burnout among physicians [16–18]. A number of socio-demographic and occupational factors appear to relate to physician burnout. For example, high workloads, lack of job control, poor work-life balance, and loss of support from colleagues have each been associated with physician burnout [19–24]. Some studies have reported an increased risk of burnout among younger physicians [24–26]. Female physicians also seem to be more likely to experience burnout than their male counterparts [27–29]. It is indicated that burnout has grave repercussions on physicians’ physical and mental health and well-being [19, 30], as well as their job performance [31–33]. More importantly, physicians experiencing burnout are more likely to be dissatisfied with their jobs and to consider leaving [34–36], which in turn threatens health workforce retention particularly in low- and middle-income countries (LMICs) with limited human resources for health. Burnout among psychiatrists has also been documented in many countries [37–41]. For example, 36.9% of psychiatrists in the US showed burnout symptoms in 2017 [17]; the prevalence rate of burnout among psychiatrists in Japan was 40% in 2016 [42]. However, most studies of burnout among psychiatrists to date were conducted in high-income countries (HICs) [43], leaving an evidence gap with regards to burnout among psychiatrists in LMICs.

In China, a vast majority (~ 80%) of psychiatrists are working at psychiatric hospitals, whereas those working at other mental health service providers, such as general hospitals, rehabilitation hospitals, and community health centers, account for only a small percentage [8]. Psychiatric hospitals in China can be further classified into two tiers: secondary psychiatric hospitals (SPHs), which tend to be affiliated with a medium size city, county or district; and tertiary psychiatric hospitals (TPHs), which are often situated in provincial capitals or major cities. In the absence of a well-developed referral system, people with mental health conditions in China have the freedom to choose where they want to obtain specialist mental health services from, and most of them prefer TPHs [44]. Moreover, in addition to providing specialist mental health services for larger geographical areas than SPHs, TPHs are also responsible for providing guidance and support to all other mental health service providers, including SPHs, in the corresponding areas [45]. Therefore, Chinese psychiatrists working at TPHs are often burdened with much heavier workloads and faced with an even grimmer crisis of burnout [46, 47].

Given the importance of burnout and job dissatisfaction to workforce retention and the central role that TPHs play in China’s mental health care system, there is a strong imperative to investigate the prevalence of burnout and job dissatisfaction among psychiatrists at TPHs in China, as well as the risk factors for burnout and job dissatisfaction. Yet, to our knowledge, there have been no nationwide studies in China to date that have achieved these purposes. Therefore, this nationwide study aimed to investigate the prevalence of burnout and job dissatisfaction among psychiatrists at TPHs in China, to identify socio-demographic and occupational factors associated with burnout and job dissatisfaction, and to examine the relationship between burnout and job dissatisfaction among psychiatrists.

## Methods

### Study design and participants

We conducted a nationwide, cross-sectional survey in March 2019 to investigate the prevalence of burnout and job dissatisfaction among psychiatrists at TPHs in China. All psychiatrists (*n* = 6986) from 41 TPHs in China were invited to participate in our survey. In total, there were 40,435 psychiatrists in China by the end of 2018, so approximately one sixth psychiatrists in China were included in our survey. To cover all the psychiatrists working at TPHs in China, we collaborated with each province’s Health Commission to issue a notice about this survey to all the TPHs situated in the corresponding province, and then the hospital administrators of these TPHs organized and facilitated the psychiatrists working at their hospitals to participate in this survey. Each psychiatrist completed a smartphone-based questionnaire anonymously and voluntarily throughout the process. All research data were de-identified and stored in a secure way to protect confidentiality. The research protocol was approved by the Ethics Committee of Chaohu Hospital of Anhui Medical University (No.201903-kyxm-02) and an electronic consent form was obtained from each participant.

### Questionnaires

The questionnaire consisted of three parts. Clear instructions were provided to participants before each section. Part 1 involved socio-demographic and occupational characteristics, i.e., age, gender (male or female), marital status (single, married, or other), site of practice (Eastern China, Central China, Western China, and Northeast China), years of practice, monthly pay in previous year, working hours per week, and having a leadership role or not.

In Part 2, the Maslach Burnout Inventory-Human Service Survey (MBI-HSS) [48] was used to measure burnout among psychiatrists in China. The Chinese version of MBI-HSS has already been validated in many studies [49–51]. It is a 22-item scale scoring the following three domains of burnout: emotional exhaustion (EE), involving nine items; depersonalization (DP), involving five items; and reduced personal accomplishment (PA), involving eight items. These items are scored on a 7-point scale from 0 to 6 according to the frequency of symptoms. Participants with high EE (≥27) or DP (≥10) scores were defined as having burnout [50, 52–55]. In our study, the Chinese version of MBI-HSS had sound face and content validity and the Cronbach’s alpha was 0.838.

In Part 3, the short version of the Minnesota Satisfaction Questionnaire (MSQ) was used to measure job satisfaction among psychiatrists in China. The Chinese version of MSQ has been widely used and demonstrated sound reliability and validity [56, 57]. MSQ has twenty items scored on a 5-point scale ranging from 1, very dissatisfied, to 5, very satisfied. The average score of these twenty items was calculated for each participant and participants with an average score of ≥3 were defined as being satisfied with their jobs in general [58]. In addition, MSQ includes two subscales: (1) intrinsic job satisfaction, which refers to whether people feel satisfied with the factors related to the nature of their jobs; and (2) extrinsic job satisfaction, which refers to whether people feel satisfied with the factors related to the working conditions that are external to their jobs [59]. The subscale scores were calculated as the average scores of subscale items, and participants with an average subscale score of ≥3 were defined as being satisfied with intrinsic or extrinsic factors of their jobs. The Chinese version of MSQ demonstrated sound reliability in our study, with the Cronbach’s alpha being 0.952.

### Statistical analysis

Continuous variables with normal distribution were reported as mean (standard deviation [SD]), and differences between groups were compared using t-tests. Continuous variables with skewed distribution were reported as median (interquartile range [IQR]) and compared using the Mann–Whitney U test. Categorical variables were presented as the number (percentage) and compared using chi-square test or unidirectional ordered chi-square test. Multivariate logistic regression was conducted to explore potential risk factors of burnout and job satisfaction. Odds ratios (ORs) for different variables and corresponding 95% confidence intervals (CIs) were reported. Statistical analyses were performed using SAS 9.4 (SAS Institute, Cary, NC, USA). Two-tailed *p* values of less than 0.05 were considered statistically significant.

## Results

### Socio-demographic and occupational characteristics of respondents

In total, there were 6986 psychiatrists working at 41 TPHs in China in 2019, and they were all invited to participate in this study. Of them, 4520 psychiatrists completed the questionnaire (completion rate, 64.7%). Table 1 shows data on their socio-demographic and occupational characteristics. 2626 respondents (58.1%) were female, and 2207 (48.8%) were aged between 30 and 39. Most respondents (81.2%) were married. About two thirds had a bachelor’s degree or less. 992 respondents (44.5%) had been practicing psychiatry for less than 5 years. 954 respondents (21.1%) had leadership roles in their workplaces, with males more likely (28.7%; 543/1892 vs 15.6%; 1349/2628; *P <* .001). The median (IQR) monthly pay of our sample was 7000 (5000–10,000) RMB, and males reported significantly higher monthly pay compared with their female counterparts (*P* = .003). The mean (SD) working hours per week was 53.0 (16.1) hours, with 4259 (94.2%) reporting for more than 40 h per week, and males reported significantly longer working hours than their female counterparts (*P* = .012).

**Table 1:** Socio-demographic and occupational characteristics of 4520 psychiatrists in China who participated in the study, n(%)

Characteristic	All (*n* = 4520)	Male (*n* = 1892)	Female (*n* = 2628)	P
Gender				
Male	1892 (41.9)	–	–	–
Female	2628 (58.1)	–	–	–
Site of practice				
Eastern China	1631 (36.1)	653 (34.5)	978 (37.2)	.003
Central China	948 (21.0)	423 (22.4)	525 (20.0)	
Western China	1197 (26.5)	472 (24.9)	725 (27.6)	
Northeast China	744 (16.5)	344 (18.2)	400 (15.2)	
Age, years				
≤ 29	602 (13.3)	163 (8.6)	439 (16.7)	<.001
30–39	2207 (48.8)	844 (44.6)	1363 (51.9)	
40–49	1082 (23.9)	510 (27.0)	572 (21.8)	
≥ 50	629 (13.9)	375 (19.8)	254 (9.7)	
Marital status				
Married	3668 (81.2)	1610 (85.1)	2058 (78.3)	<.001
Single	690 (15.3)	221 (11.7)	469 (17.8)	
Other	162 (3.6)	61 (3.2)	101 (3.8)	
Education				
Associate degree or less	123 (2.7)	69 (3.6)	54 (2.1)	<.001
Bachelor’s degree	2866 (63.4)	1287 (68.0)	1579 (60.1)	
Master’s degree	1257 (27.8)	425 (22.5)	832 (31.7)	
Doctorate degree	274 (6.1)	111 (5.9)	163 (6.2)	
Years of practice, years				
≤ 5	992 (21.9)	308 (16.3)	684 (26.0)	<.001
6–10	1023 (22.6)	358 (18.9)	665 (25.3)	
11–20	1405 (31.1)	634 (33.5)	771 (29.3)	
≥ 21	1100 (24.3)	592 (31.3)	508 (19.3)	
Leadership role				
Yes	954 (21.1)	543 (28.7)	1349 (15.6)	<.001
No	3566 (78.9)	411 (71.3)	2217 (84.4)	
Monthly pay in previous year, RMB^a^				
< 5000	720 (16.5)	278 (14.7)	442 (16.8)	<.001
5000–7999	1604 (36.7)	636 (33.6)	968 (36.8)	
8000–11,999	1395 (31.9)	615 (32.5)	780 (29.7)	
≥ 12,000	649 (14.9)	310 (16.4)	339 (12.9)	
Working hours per week, hours				
≤ 40	261 (5.8)	124 (6.6)	137 (5.2)	.026
41–50	1976 (43.7)	794 (42.0)	1182 (45.0)	
51–60	781 (17.3)	314 (16.6)	467 (17.8)	
≥ 61	1502 (33.2)	660 (34.9)	842 (32.0)	

a. Ratio of US dollar to Chinese yuan (RMB) = 6.47

### Prevalence of burnout and job dissatisfaction

Table 2 shows data on burnout and job dissatisfaction in our sample. 1146 (25.4%) respondents reported a high level of EE, 1485 (32.9%) reported a high level of DP, and 935 (20.7%) reported a low level of PA. Overall, 1735 (38.4%) respondents met the criteria of burnout. Moreover, males in our sample had a slightly higher rate of burnout (39.6%; 750/1892) compared with females (37.5%; 985/2628), but the difference was not statistically significant (*P* = .141).

**Table 2:** Prevalence of burnout and job satisfaction in a sample of 4520 psychiatrists in China, n(%)

	All (*n* = 4520)	Male (*n* = 1892)	Female (*n* = 2628)	P
Burnout				
Yes	1735 (38.4)	750 (39.6)	985 (37.5)	.141
No	2785 (61.6)	1142 (60.4)	1643 (62.5)	
Emotional exhaustion				
Low	2419 (53.5)	1019 (53.9)	1400 (53.3)	.634
Mid	955 (21.1)	387 (20.5)	568 (21.6)	
High	1146 (25.4)	486 (25.7)	660 (25.1)	
Depersonalization				
Low	1538 (34.0)	606 (32.0)	932 (35.5)	.029
Mid	1497 (33.1)	630 (33.3)	867 (33.0)	
High	1485 (32.9)	656 (34.7)	829 (31.5)	
Personal accomplishment				
Low	2555 (56.5)	1099 (58.1)	1456 (55.4)	.146
Mid	1030 (22.8)	407 (21.5)	623 (23.7)	
High	935 (20.7)	386 (20.4)	549 (20.9)	
General Job Satisfaction				
Yes	2911 (64.4)	1127 (59.6)	1784 (67.9)	<.001
No	1609 (35.6)	765 (40.4)	844 (32.1)	
Intrinsic Job Satisfaction				
Yes	3136 (69.4)	1212 (64.1)	1924 (73.2)	<.001
No	1384 (30.6)	680 (35.9)	704 (26.8)	
Extrinsic Job Satisfaction				
Yes	2362 (52.3)	983 (52.0)	1175 (44.7)	<.001
No	2158 (47.7)	909 (48.0)	1453 (55.3)	

Overall, 1609 respondents (35.6%) were dissatisfied with their jobs in general, and males reported a higher rate of general job dissatisfaction than females (59.6%; 765/1892 vs 67.9%; 844/2628; *P* < .001). Interestingly, males were more likely to be dissatisfied with intrinsic factors of their jobs than females (35.9%; 680/1892 vs 26.8%; 704/2628; P < .001) but less likely to be dissatisfied with extrinsic factors than females (48.0%; 909/1892 vs 55.3%; 1454/2628; P < .001).

### Factors associated with burnout and job dissatisfaction

We examined the associations between respondent characteristics and burnout (Table 3, also see **Supplementary Table 1** in Additional File 1). It was found that being male (OR 1.20, 95% CI 1.05–1.37), 11–20 years of practice (OR 1.42, 95% CI 1.10–1.84; reference: ≤5 years), having no leadership role (OR 1.60, 95% CI 1.32–1.95), and longer working hours per week (41–50 h: OR 1.47, 95% CI 1.08–2.01; 51–60 h: OR 2.39, 95% CI 1.71–3.34; ≥61 h: OR 3.09, 95% CI 2.25–4.25; reference: ≤40 h) were significantly associated with burnout. There were no significant associations between monthly pay and burnout (5000–7999 RMB: OR 0.90, 95% CI 0.74–1.09; 8000–11,999 RMB: OR 0.88, 95% 0.71–1.10; ≥12,000 RMB: OR 0.77, 95% CI 0.58–1.03; reference: < 5000 RMB) after adjusting for other variables.

**Table 3:** Effects of respondent characteristics on burnout and general job satisfaction in a sample of 4520 psychiatrists in China

	Burnout			General Job Satisfaction		
	All	Male	Female	All	Male	Female
	odds ratio (95% CI)			odds ratio (95% CI)		
Gender						
Female	Reference	–	–	Reference	–	–
Male	1.20 (1.05–1.37)**	–	–	0.66 (0.58–0.76)***	–	–
Site of practice						
Eastern China	Reference	Reference	Reference	Reference	Reference	Reference
Central China	0.98 (0.82–1.17)	0.88 (0.67–1.16)	1.07 (0.84–1.35)	0.99 (0.83–1.19)	1.01 (0.77–1.32)	0.97 (0.76–1.24)
Western China	0.92 (0.77–1.10)	0.99 (0.75–1.31)	0.89 (0.71–1.12)	0.98 (0.82–1.17)	1.02 (0.77–1.34)	0.93 (0.73–1.18)
Northeast China	0.83 (0.67–1.04)	0.93 (0.67–1.3)	0.74 (0.55–1.00)	1.29 (1.03–1.61)*	1.45 (1.05–2.01)*	1.14 (0.85–1.55)
Age, years						
≤ 29	Reference	Reference	Reference	Reference	Reference	Reference
30–39	1.09 (0.83–1.42)	1.00 (0.63–1.61)	1.14 (0.82–1.58)	0.89 (0.67–1.17)	1.00 (0.63–1.61)	0.82 (0.58–1.15)
40–49	1.07 (0.75–1.51)	1.02 (0.57–1.81)	1.10 (0.70–1.72)	0.78 (0.54–1.11)	1.00 (0.57–1.77)	0.62 (0.39–0.99)*
≥ 50	0.76 (0.49–1.18)	0.62 (0.32–1.22)	0.94 (0.51–1.72)	0.92 (0.59–1.43)	1.17 (0.60–2.27)	0.76 (0.41–1.42)
Marital status						
Married	Reference	Reference	Reference	Reference	Reference	Reference
Single	1.19 (0.96–1.47)	1.16 (0.81–1.66)	1.23 (0.95–1.59)	0.88 (0.71–1.10)	0.91 (0.63–1.30)	0.87 (0.67–1.14)
Other	1.28 (0.91–1.79)	1.15 (0.66–2.01)	1.36 (0.89–2.08)	0.75 (0.54–1.05)	0.73 (0.43–1.26)	0.76 (0.49–1.17)
Education						
Associate degree or less	Reference	Reference	Reference	Reference	Reference	Reference
Bachelor’s degree	0.97 (0.63–1.47)	0.98 (0.57–1.71)	0.98 (0.51–1.91)	0.75 (0.50–1.14)	0.95 (0.56–1.61)	0.55 (0.28–1.10)
Master’s degree	1.01 (0.64–1.58)	1.05 (0.57–1.93)	1.00 (0.50–2.00)	0.73 (0.47–1.13)	0.93 (0.52–1.68)	0.53 (0.26–1.08)
Doctorate degree	0.73 (0.43–1.22)	0.75 (0.36–1.56)	0.72 (0.33–1.56)	0.87 (0.52–1.46)	1.08 (0.53–2.19)	0.65 (0.29–1.46)
Years of practice, years						
≤ 5	Reference	Reference	Reference	Reference	Reference	Reference
6–10	1.14 (0.90–1.45)	1.45 (0.98–2.15)	1.00 (0.74–1.34)	0.77 (0.60–0.98)*	0.68 (0.46–1.01)	0.85 (0.62–1.16)
11–20	1.42 (1.10–1.84)**	1.59 (1.06–2.39)*	1.34 (0.96–1.86)	0.56 (0.43–0.73)***	0.54 (0.36–0.82)**	0.59 (0.42–0.83)**
≥ 21	1.17 (0.81–1.70)	1.56 (0.89–2.74)	0.94 (0.57–1.55)	0.58 (0.40–0.85)**	0.53 (0.30–0.92)*	0.66 (0.40–1.11)
Leadership role						
Yes	Reference	Reference	Reference	Reference	Reference	Reference
No	1.60 (1.32–1.95)***	1.75 (1.34–2.3)***	1.42 (1.07–1.88)*	0.54 (0.44–0.65)***	0.58 (0.44–0.75)***	0.49 (0.37–0.67)***
Monthly pay in previous year, RMB						
< 5000	Reference	Reference	Reference	Reference	Reference	Reference
5000–7999	0.90 (0.74–1.09)	1.02 (0.75–1.39)	0.81 (0.63–1.04)	1.06 (0.87–1.29)	0.94 (0.69–1.27)	1.14 (0.88–1.48)
8000–11,999	0.88 (0.71–1.10)	0.86 (0.61–1.22)	0.89 (0.67–1.18)	1.50 (1.20–1.87)***	1.51 (1.07–2.13)*	1.47 (1.10–1.97)**
≥ 12,000	0.77 (0.58–1.03)	0.75 (0.49–1.16)	0.79 (0.54–1.14)	1.92 (1.44–2.56)***	1.59 (1.04–2.43)*	2.32 (1.57–3.45)***
Working hours per week, hours						
≤ 40	Reference	Reference	Reference	Reference	Reference	Reference
41–50	1.47 (1.08–2.01)	1.35(0.86–2.12)	1.57 (1.01–2.43)*	0.87 (0.65–1.16)	1.01 (0.67–1.52)	0.72 (0.47–1.10)
51–60	2.39 (1.71–3.34)***	2.23(1.37–3.63)**	2.56 (1.61–4.06)***	0.70 (0.51–0.96)*	0.82 (0.52–1.29)	0.58 (0.37–0.92)*
≥ 61	3.09 (2.25–4.25)***	2.97(1.87–4.71)***	3.22 (2.07–5.03)***	0.60 (0.44–0.81)***	0.72 (0.47–1.1)	0.49 (0.31–0.75)**

Notes: * *P* < .05; ** *P* < .01; *** P < .001

The associations between socio-demographic characteristics and job satisfaction were also examined (Table 3, also see **Supplementary Table 2** in Additional File 1). It was found that being male (OR 0.66, 95% CI 0.58–0.76), working in Northeast China (OR 1.29, 95% CI 1.03–1.61; reference: Eastern China), more years of practice (6–10 years: OR 0.77, 95% CI 0.6–0.98; 11–20 years: OR 0.56, 95% CI 0.43–0.73; ≥21 years: OR 0.58, 95% CI 0.4–0.85; reference: ≤5 years), having no leadership role (OR 0.54, 95% CI 0.44–0.65), lower monthly pay (8000–11,999 RMB: OR 1.50, 95% CI 1.20–1.87; ≥12,000 RMB: OR 1.92, 95% CI 1.44–2.56; reference: < 5000 RMB), and longer working hours per week (51–60 h: OR 0.70, 95% CI 0.51–0.96; ≥61 h: OR 0.60, 95% CI 0.44–0.81; reference: ≤40 h) were significantly associated with job dissatisfaction in general.

### Relationship between burnout and job satisfaction

**Fig. 1:**
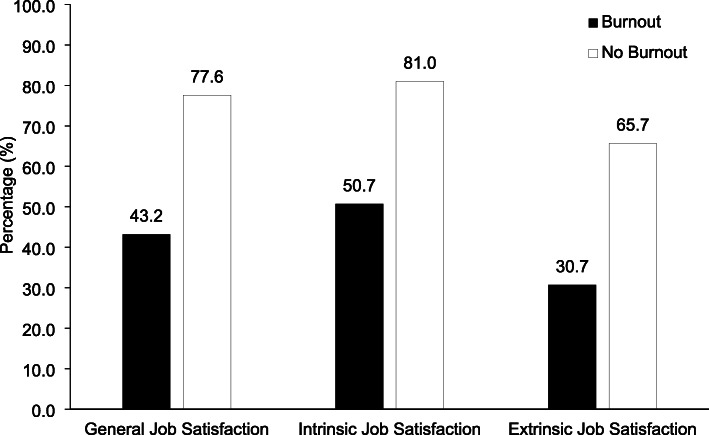
Prevalence of job satisfaction among respondents with or without burnout in a sample of 4520 psychiatrists in China

The prevalence of job satisfaction among respondents with and without burnout is shown in Fig. 1. Seven hundred fifty respondents (43.2%) with burnout were satisfied with their jobs in general, while 2140 respondents (77.6%) without burnout were satisfied. As shown in Table 4, burnout was negatively associated with general job satisfaction (OR 0.23, 95% CI 0.20–0.26, *P* < .001), after adjustment for gender, age, marital status, education, site of practice, years of practice, having a leadership role or not, monthly pay, and working hours per week. Similarly, respondents with burnout were significantly more likely to report dissatisfaction with intrinsic factors of their jobs (OR 0.25, 95% CI 0.21–0.28, *P* < .001), as well as extrinsic factors (OR 0.25, 95% CI 0.22–0.29, *P* < .001).

**Table 4:** Effects of burnout on job satisfaction in a sample of 4520 psychiatrists in China

	Job Satisfaction								
	General Job Satisfaction			Intrinsic Job Satisfaction			Extrinsic Job Satisfaction		
	All^a^	Male^b^	Female^b^	All^a^	Male^b^	Female^b^	All^a^	Male^b^	Female^b^
	odds ratio (95% CI)			odds ratio (95% CI)			odds ratio (95% CI)		
Burnout									
No	Reference	Reference	Reference	Reference	Reference	Reference	Reference	Reference	Reference
Yes	0.23 (0.20–0.26)	0.23 (0.19–0.29)	0.22 (0.18–0.26)	0.25 (0.21–0.28)	0.23 (0.18–0.28)	0.25 (0.21–0.30)	0.25 (0.22–0.29)	0.25 (0.21–0.31)	0.24 (0.2–0.30)
Emotional exhaustion									
Low	Reference	Reference	Reference	Reference	Reference	Reference	Reference	Reference	Reference
Mid	0.33 (0.28–0.4)	0.35 (0.27–0.45)	0.32 (0.26–0.41)	0.33 (0.28–0.38)	0.34 (0.26–0.44)	0.32 (0.25–0.39)	0.38 (0.32–0.46)	0.38 (0.29–0.49)	0.39 (0.30–0.50)
High	0.12 (0.10–0.14)	0.11 (0.08–0.14)	0.12 (0.10–0.16)	0.12 (0.10–0.15)	0.11 (0.08–0.14)	0.13 (0.10–0.16)	0.15 (0.12–0.18)	0.14 (0.11–0.19)	0.15 (0.12–0.19)
Depersonalization									
Low	Reference	Reference	Reference	Reference	Reference	Reference	Reference	Reference	Reference
Mid	0.5 (0.42–0.60)	0.48 (0.37–0.62)	0.51 (0.40–0.65)	0.5 (0.43–0.58)	0.52 (0.41–0.66)	0.47 (0.39–0.58)	0.5 (0.41–0.60)	0.45 (0.34–0.59)	0.53 (0.42–0.69)
High	0.19 (0.16–0.22)	0.19 (0.14–0.24)	0.18 (0.14–0.23)	0.2 (0.17–0.24)	0.20 (0.15–0.25)	0.20 (0.16–0.25)	0.20 (0.17–0.24)	0.20 (0.15–0.26)	0.20 (0.15–0.25)
Personal accomplishment									
Low	Reference	Reference	Reference	Reference	Reference	Reference	Reference	Reference	Reference
Mid	2.46 (2.09–2.90)	2.96 (2.29–3.82)	2.12 (1.71–2.64)	1.97 (1.69–2.29)	2.07 (1.62–2.63)	1.92 (1.57–2.35)	2.73 (2.29–3.26)	3.18 (2.43–4.16)	2.37 (1.87–2.99)
High	5.57 (4.51–6.88)	6.02 (4.42–8.22)	5.43 (4.06–7.27)	3.76 (3.16–4.48)	3.79 (2.91–4.94)	3.91 (3.08–4.95)	6.5 (5.12–8.24)	6.06 (4.36–8.44)	7.27 (5.13–10.31)

a. After adjustment for gender, site of practice, age, marital status, education, years of practice, having a leadership role or not, monthly pay, and working hours per week

b. After adjustment for site of practice, age, marital status, education, years of practice, having a leadership role or not, monthly pay, and working hours per week

Notes: The Ps values of all results were < .001

## Discussion

Our study was the first nationwide survey that comprehensively investigated the prevalence of burnout and job satisfaction among psychiatrists in China. The sample of > 4000 psychiatrists, covering all the TPHs in China, represents the largest study of psychiatrist burnout in the literature.

We found a high rate of burnout (38.4%) among psychiatrists working at TPHs in China. This rate was concordant with those reported in HICs such as the US (36.9%) [17] and Japan (40.0%) [42]. More specifically, our study found that 25.4% of psychiatrists in China had high levels of EE, 32.9% had high levels of DP, and 20.7% had low levels of PA. These rates were also consistent with the overall estimated pooled prevalence for high levels of EE, higher levels of DP, and low levels of PA among mental health professionals [43]. There are many causes that may lead to burnout among psychiatrists in China, such as widespread stigma towards mental illness [60], heavy workload [61], low salary [62], and poor physician-patient relationships [63–66]. Interestingly, although the prevalence of burnout among psychiatrists in China is comparable to those in HICs, the percentage of job satisfaction among psychiatrists in China is much lower than those reported in HICs [67–69]. For example, 88% of Australian psychiatrists were satisfied with their work and proud of their profession [69]. In contrast, only 64.4% of respondents in our study were satisfied with their jobs in general. For those experiencing burnout, the percentage of psychiatrists reporting job satisfaction was even lower (43.2%). Despite the fact that burnout and job dissatisfaction are closely interrelated, this finding supports the view that burnout and job dissatisfaction are two distinct indicators of job morale with different etiologies [70].

Our study suggests that male psychiatrists in China seem to be at higher risk of burnout and job dissatisfaction. This is inconsistent with the findings from HICs, e.g., the US [39], Austria [71], and France [72]. One possible interpretation is that psychiatrists in China often have to live with unsatisfactory payment [62] and deep-rooted stigmatization towards mental health professionals [60], which conflicts with the expectations attached to males in China to gain more wealth and higher status. We also found that early-career psychiatrists reported lower rates of burnout and job dissatisfaction, which was again inconsistent with the findings in North American psychiatrists [39] and European psychiatrists [73]. There may be two explanations. Firstly, because of relatively low tuition costs, medical students in China rarely reply on student loans to finish their medical schools. So, unlike many early-career psychiatrists in HICs, their counterparts in China are often not burdened with medical school debts after graduation and are therefore faced with less financial stress. Indeed, several studies have found strong associations between medical school debts and burnout among early-career physicians [29, 74]. Secondly, most psychiatrists in China start residency training immediately after receiving medical bachelor’s degrees, as demonstrated in Table 1. Therefore, early-career psychiatrists in China are usually much younger than those in many HICs, which makes them to some extent freed from household debts, as well as other family responsibilities.

Till now, there have been an enormous body of evidence suggesting that longer working hours [72, 73, 75, 76] and less control over one’s job [39] can lead to burnout and job dissatisfaction. Similarly, our study also reported that longer working hours were significantly associated with both burnout and job dissatisfaction. Regarding job control, it should be noted that our study asked its respondents whether they had leadership roles in their workplaces instead of asking directly how much control they had over their jobs. However, team leaders are often considered to have more control over their jobs. Accordingly, our data suggest that having leadership roles in the workplace was significantly associated with less burnout and more job satisfaction among psychiatrists in China.

It is often reported that psychiatrists have a lower rate of burnout compared with other specialists [16, 17, 20]. Our study also showed a lower rate of burnout (38.4%) among psychiatrists than those among other specialists, such as neurologists (53.2%) [77], anesthesiologists (69%) [49], and oncologists (51%) [50], in China. There are several possible explanations for this finding. Firstly, psychiatrists may be more aware of their own emotional and psychological needs than other specialists. Also, psychiatrists may be more skilled at stress relieving and have better access to mental health services.

A large body of research has suggested that burnout and job dissatisfaction among health workers is associated with an increased likelihood of leaving their jobs [34–36]. Therefore, a high rate of burnout and job dissatisfaction that we found among psychiatrists in China will evidently complicate the current challenges of workforce development in mental health. In order to mitigate burnout and job dissatisfaction among psychiatrists in China, a systemic strategy attempting to tackle with various factors on different levels is needed [78]. First, on the organizational level, staff care and wellbeing programs, such as mindfulness, stress management, and small group discussions [79], should be developed and provided for psychiatrists. A culture of mutual support should also be created and advocated in mental health care facilities [80]. Second, on the national level, new investments should be encouraged in mental health care and particularly for mental health workforce retention. With low salary as an important contributor to job dissatisfaction, the salary and compensation system at psychiatric hospitals needs to be reformed. To better remunerate Chinese psychiatrists, a workload-based salary scheme should be adopted [62]. Finally, on the societal level, it is also of great importance to destigmatize mental health problems and to improve public attitudes towards psychiatry and psychiatrists [81].

Our study had several limitations. First, we utilized self-report measures of burnout and job satisfaction. Although both MBI and MSQ were well validated in China and in other countries, their self-report nature makes their validity still questionable. Second, our study included psychiatrists working in psychiatric hospitals but not those working in other health care settings such as primary care facilities. However, according to the official data from China’s National Health Commission, 80.2% of mental health professionals in China worked in psychiatric hospitals [8]. Therefore, our study can still stand out as being of crucial importance to landscape and investigate burnout and job satisfaction among psychiatrists in China. Third, the cross-sectional survey method does not allow assessment of the direction of effect for the associations described in this study. Future studies are needed to investigate prospectively the effects of relevant factors on psychiatrists’ burnout and job dissatisfaction.

## Conclusions

In summary, our study suggested that psychiatrists in China experienced a high rate of burnout and job dissatisfaction. Although the prevalence of burnout among them was similar with those reported in HICs, a much lower rate of job satisfaction was found in psychiatrists in China. Although China’s government has made much effort to attract and recruit new psychiatrists since the release of the *2015–2020 National Mental Health Work Plan*, our study suggests that at least as much attention should be paid to the crisis of burnout and job satisfaction in psychiatrists in China which will sabotage workforce retention. In order to preserve and strengthen the mental health workforce, proactive measures are urgently needed to mitigate burnout and job dissatisfaction among psychiatrists in China.

## Supplementary Information

**Additional File 1: Supplementary Table 1.** Effects of respondent characteristics on emotional exhaustion, depersonalization, and personal accomplishment measured by the Maslach Burnout Inventory-Human Service Survey in a sample of 4520 psychiatrists in China. **Supplementary Table 2.** Effects of respondent characteristics on intrinsic and extrinsic job satisfaction measured by the Minnesota Satisfaction Questionnaire in a sample of 4520 psychiatrists in China

## Data Availability

Additional data available from the corresponding author at jimingzhu@tsinghua.edu.cn.

## Abbreviations

HIC: high-income country
LMIC: low- and middle-income countries
SPH: secondary psychiatric hospital
TPH: tertiary psychiatric hospital
MBI-HSS: Maslach Burnout Inventory-Human Service Survey
EE: emotional exhaustion
DP: depersonalization
PA: personal accomplishment
MSQ: Minnesota Satisfaction Questionnaire
SD: standard deviation
IQR: interquartile range
OR: odds ratio
CI: confidence interval
