# Supplementary material for: Burnout and job satisfaction of psychiatrists in China: a nationwide survey
Source: BMC Psychiatry. 2021 Nov 24;21:593. doi: 10.1186/s12888-021-03568-6 (PMC8612106; doi:10.1186/s12888-021-03568-6)
Supplement: Supplementary file 1 — Additional File 1: Supplementary Table 1. Effects of respondent characteristics on emotional exhaustion, depersonalization, and personal accomplishment measured by the Maslach Burnout Inventory-Human Service Survey in a sample of 4520 psychiatrists in China. Supplementary Table 2. Effects of respondent characteristics on intrinsic and extrinsic job satisfaction measured by the Minnesota Satisfaction Questionnaire in a sample of 4520 psychiatrists in China [file 12888_2021_3568_MOESM1_ESM.docx]

**Supplemental Materials**

eTable 1. Effects of respondent characteristics on emotional exhaustion, depersonalization, and personal accomplishment measured by the Maslach Burnout Inventory-Human Service Survey in a sample of 4520 psychiatrists in China

eTable 2. Effects of respondent characteristics on intrinsic and extrinsic job satisfaction measured by the Minnesota Satisfaction Questionnaire in a sample of 4520 psychiatrists in China

**eTable 1. Effects of respondent characteristics on emotional exhaustion, depersonalization, and personal accomplishment measured by the Maslach Burnout Inventory-Human Service Survey in a sample of 4520 psychiatrists in China**

|  | Emotional exhaustion | | | Depersonalization | | | Personal accomplishment | | |
| --- | --- | --- | --- | --- | --- | --- | --- | --- | --- |
|  | All | Male | Female | All | Male | Female | All | Male | Female |
|  | odds ratio (95% CI) | | | odds ratio (95% CI) | | |  |  |  |
| Gender |  |  |  |  |  |  |  |  |  |
| Female | Reference | - | - | Reference | - | - | Reference | - | - |
| Male | 1.12 (0.97-1.29) | - | - | 1.27 (1.11-1.45)*** | - | - | 0.81 (0.71-0.92)** |  |  |
| Site of practice |  |  |  |  |  |  |  |  |  |
| Eastern China | Reference | Reference | Reference | Reference | Reference | Reference | Reference | Reference | Reference |
| Central China | 1.01 (0.83-1.23) | 0.84 (0.61-1.14) | 1.17 (0.9-1.52) | 0.95 (0.79-1.14) | 0.95 (0.72-1.26) | 0.96 (0.75-1.22) | 1.17 (0.99-1.4) | 0.98 (0.75-1.29) | 1.35 (1.07-1.71)* |
| Western China | 1.07 (0.88-1.31) | 1.17 (0.86-1.6) | 1.03 (0.8-1.33) | 0.83 (0.69-0.99)* | 0.94 (0.7-1.25) | 0.76 (0.59-0.96)* | 1.02 (0.86-1.22) | 1.08 (0.82-1.42) | 0.99 (0.79-1.25) |
| Northeast China | 0.94 (0.73-1.2) | 1.00 (0.69-1.45) | 0.86 (0.61-1.2) | 0.82 (0.65-1.02) | 0.95 (0.68-1.34) | 0.70 (0.51-0.95)* | 1.26 (1.02-1.56)* | 1.06 (0.77-1.47) | 1.47 (1.11-1.95)** |
| Age, years |  |  |  |  |  |  |  |  |  |
| ≤29 | Reference | Reference | Reference | Reference | Reference | Reference | Reference | Reference | Reference |
| 30-39 | 1.05 (0.78-1.42) | 0.91 (0.54-1.53) | 1.12 (0.77-1.62) | 1.09 (0.83-1.43) | 1.06 (0.65-1.71) | 1.14 (0.81-1.59) | 1.00 (0.77-1.3) | 0.86 0.53-1.38) | 1.06 (0.77-1.46) |
| 40-49 | 1.09 (0.74-1.6) | 0.82 (0.44-1.54) | 1.33 (0.81-2.18) | 0.99 (0.69-1.41) | 0.98 (0.55-1.75) | 1.01 (0.64-1.61) | 1.04 (0.74-1.46) | 0.81 (0.46-1.45) | 1.13 (0.73-1.75) |
| ≥50 | 0.77 (0.47-1.27) | 0.44 (0.21-0.95)* | 1.33 (0.67-2.65) | 0.68 (0.43-1.08) | 0.58 (0.29-1.17) | 0.85 (0.45-1.61) | 1.29 (0.84-1.97) | 1.11 (0.57-2.16) | 1.26 (0.71-2.24) |
| Marital status |  |  |  |  |  |  |  |  |  |
| Married | Reference | Reference | Reference | Reference | Reference | Reference | Reference | Reference | Reference |
| Single | 1.20 (0.95-1.51) | 1.17 (0.79-1.73) | 1.24 (0.93-1.66) | 1.22 (0.98-1.51) | 1.22 (0.85-1.76) | 1.23 (0.94-1.61) | 0.97 (0.79-1.19) | 0.84 (0.58-1.21) | 1.03 (0.8-1.33) |
| Other | 1.46 (1.02-2.09)* | 1.01 (0.54-1.89) | 1.81 (1.16-2.83)** | 1.23 (0.87-1.75) | 1.23 (0.7-2.16) | 1.24 (0.79-1.94) | 1.06 (0.76-1.47) | 1.60 (0.93-2.74) | 0.81 (0.53-1.24) |
| Education |  |  |  |  |  |  |  |  |  |
| Associate degree or less | Reference | Reference | Reference | Reference | Reference | Reference | Reference | Reference | Reference |
| Bachelor’s degree | 1.02 (0.62-1.69) | 1.06 (0.55-2.07) | 1.03 (0.47-2.24) | 1.03 (0.66-1.62) | 1.07 (0.59-1.92) | 1.03 (0.50-2.11) | 0.94 (0.64-1.39) | 1.00 (0.59-1.68) | 0.89 (0.5-1.59) |
| Master’s degree | 1.02 (0.60-1.74) | 1.09 (0.53-2.25) | 1.01 (0.45-2.27) | 1.02 (0.63-1.65) | 1.15 (0.60-2.18) | 0.95 (0.45-2.01) | 1.18 (0.78-1.78) | 1.1 (0.61-1.96) | 1.19 (0.64-2.19) |
| Doctorate degree | 0.79 (0.43-1.45) | 0.90 (0.38-2.11) | 0.73 (0.30-1.80) | 0.82 (0.47-1.42) | 0.96 (0.45-2.07) | 0.74 (0.32-1.70) | 1.65 (1.01-2.67)* | 1.71 (0.86-3.43) | 1.57 (0.78-3.15) |
| Years of practice, years |  |  |  |  |  |  |  |  |  |
| ≤5 | Reference | Reference | Reference | Reference | Reference | Reference | Reference | Reference | Reference |
| 6-10 | 1.18 (0.91-1.54) | 1.62 (1.05-2.51)* | 0.97 (0.69-1.36) | 1.15 (0.91-1.47) | 1.59 (1.06-2.36)* | 0.96 (0.70-1.30) | 0.96 (0.76-1.21) | 1.01 (0.68-1.51) | 0.94 (0.7-1.25) |
| 11-20 | 1.56 (1.17-2.07)** | 1.73 (1.09-2.72)* | 1.47 (1.02-2.13)* | 1.34 (1.03-1.74)* | 1.64 (1.08-2.49)* | 1.18 (0.84-1.65) | 1.03 (0.8-1.32) | 1.18 (0.78-1.78) | 0.96 (0.69-1.32) |
| ≥21 | 1.19 (0.79-1.81) | 1.90 (1.01-3.58)* | 0.79 (0.45-1.39) | 1.14 (0.78-1.67) | 1.51 (0.85-2.68) | 0.94 (0.56-1.59) | 1.13 (0.79-1.62) | 1.09 (0.62-1.89) | 1.24 (0.76-2.02) |
| Leadership role |  |  |  |  |  |  |  |  |  |
| Yes | Reference | Reference | Reference | Reference | Reference | Reference | Reference | Reference | Reference |
| No | 1.72 (1.37-2.15)*** | 1.88 (1.37-2.59)*** | 1.52 (1.10-2.10)* | 1.50 (1.22-1.84)*** | 1.57 (1.19-2.08)** | 1.40(1.03-1.89)* | 0.63 (0.52-0.75)*** | 0.62 (0.48-0.80)*** | 0.62 (0.48-0.81)*** |
| Monthly pay in previous year, RMB |  |  |  |  |  |  |  |  |  |
| <5000 | Reference | Reference | Reference | Reference | Reference | Reference | Reference | Reference | Reference |
| 5000-7999 | 1.02 (0.82-1.26) | 1.21 (0.86-1.71) | 0.90 (0.67-1.19) | 0.88 (0.72-1.07) | 0.95 (0.7-1.31) | 0.81 (0.62-1.05) | 1.03 (0.85-1.25) | 0.88 (0.64-1.21) | 1.15 (0.89-1.48) |
| 8000-11999 | 0.93 (0.72-1.18) | 0.86 (0.58-1.26) | 0.98 (0.71-1.35) | 0.83 (0.66-1.03) | 0.79 (0.56-1.13) | 0.85 (0.63-1.14) | 1.12 (0.9-1.39) | 1.12 (0.8-1.58) | 1.12 (0.84-1.48) |
| ≥12000 | 0.89 (0.65-1.23) | 0.91 (0.56-1.47) | 0.88 (0.58-1.33) | 0.72 (0.54-0.96)* | 0.71 (0.46-1.11) | 0.71 (0.49-1.05) | 1.5 (1.14-1.97)** | 1.28 (0.84-1.94) | 1.75 (1.22-2.51)** |
| Working hours per week, hours |  |  |  |  |  |  |  |  |  |
| ≤40 | Reference | Reference | Reference | Reference | Reference | Reference | Reference | Reference | Reference |
| 41-50 | 2.00 (1.32-3.04)** | 2.18 (1.16-4.11)* | 1.84 (1.05-3.23)* | 1.35 (0.97-1.87) | 1.11 (0.7-1.76) | 1.62 (1.01-2.60)* | 1.02 (0.78-1.34) | 1.13 (0.76-1.7) | 0.96 (0.66-1.39) |
| 51-60 | 3.19 (2.06-4.93)**** | 3.52 (1.81-6.84)*** | 2.99 (1.66-5.37)*** | 2.22 (1.57-3.14)*** | 1.75 (1.06-2.87)* | 2.77 (1.68-4.57)*** | 1.06 (0.79-1.43) | 1.13 (0.72-1.76) | 1.03 (0.69-1.54) |
| ≥61 | 4.50 (2.95-6.86)**** | 4.85 (2.56-9.16)*** | 4.25 (2.41-7.49)*** | 2.68 (1.93-3.74)*** | 2.20 (1.38-3.51)*** | 3.23 (2.00-5.23)*** | 0.98 (0.74-1.3) | 1.05 (0.69-1.6) | 0.95 (0.65-1.39) |

Notes: * P < .05; ** P < .01; *** P < .001

**eTable 2. Effects of respondent characteristics on intrinsic and extrinsic job satisfaction measured by the Minnesota Satisfaction Questionnaire in a sample of 4520 psychiatrists in China**

|  | Intrinsic Job Satisfaction | | | Extrinsic Job Satisfaction | | |
| --- | --- | --- | --- | --- | --- | --- |
|  | All | Male | Female | All | Male | Female |
|  | odds ratio (95% CI) | | | odds ratio (95% CI) | | |
| Gender |  |  |  |  |  |  |
| Female | Reference | - | - | Reference | - | - |
| Male | 0.60 (0.53-0.69)*** |  |  | 0.72 (0.64-0.82)*** |  |  |
| Site of practice |  |  |  |  |  |  |
| Eastern China | Reference | Reference | Reference | Reference | Reference | Reference |
| Central China | 1.13 (0.94-1.37) | 1.09 (0.82-1.43) | 1.19 (0.91-1.55) | 0.87 (0.73-1.04) | 0.93 (0.71-1.21) | 0.82 (0.65-1.03) |
| Western China | 0.91 (0.76-1.09) | 1.01 (0.76-1.34) | 0.84 (0.65-1.07) | 1.03 (0.87-1.23) | 1.11 (0.84-1.46) | 0.96 (0.76-1.20) |
| Northeast China | 1.35 (1.07-1.70)* | 1.39 (1.00-1.94) | 1.30 (0.95-1.79) | 1.30 (1.05-1.61)* | 1.42 (1.03-1.96)* | 1.21 (0.91-1.61) |
| Age, years |  |  |  |  |  |  |
| ≤29 | Reference | Reference | Reference | Reference | Reference | Reference |
| 30-39 | 0.94 (0.71-1.25) | 0.82 (0.51-1.33) | 0.98 (0.68-1.41) | 0.86 (0.66-1.12) | 1.29 (0.81-2.04) | 0.72 (0.52-1.00) |
| 40-49 | 0.70 (0.48-1.01) | 0.73 (0.41-1.31) | 0.60 (0.37-0.97)* | 0.62 (0.44-0.87)** | 1.04 (0.59-1.82) | 0.47 (0.30-0.73)*** |
| ≥50 | 0.80 (0.50-1.26) | 0.84 (0.42-1.66) | 0.66 (0.34-1.27) | 0.8 (0.52-1.22) | 1.23 (0.64-2.37) | 0.66 (0.37-1.18) |
| Marital status |  |  |  |  |  |  |
| Married |  |  |  |  |  |  |
| Single | 0.94 (0.75-1.17) | 0.83 (0.58-1.19) | 1.02 (0.76-1.36) | 0.91 (0.74-1.12) | 1.01 (0.71-1.44) | 0.87 (0.67-1.13) |
| Other | 0.98 (0.69-1.39) | 1.10 (0.63-1.92) | 0.88 (0.55-1.39) | 0.70 (0.50-0.99)** | 0.67 (0.38-1.19) | 0.73 (0.48-1.11) |
| Education |  |  |  |  |  |  |
| Associate degree or less | Reference | Reference | Reference | Reference | Reference | Reference |
| Bachelor’s degree | 0.84 (0.55-1.29) | 1.06 (0.62-1.81) | 0.60 (0.29-1.23) | 0.89 (0.60-1.32) | 1.11 (0.66-1.88) | 0.69 (0.38-1.25) |
| Master’s degree | 0.74 (0.47-1.16) | 1.02 (0.57-1.85) | 0.49 (0.23-1.03) | 0.84 (0.56-1.28) | 1.13 (0.63-2.01) | 0.62 (0.33-1.17) |
| Doctorate degree | 0.94 (0.55-1.60) | 1.39 (0.67-2.89) | 0.58 (0.25-1.35) | 0.86 (0.53-1.40) | 1.14 (0.57-2.29) | 0.64 (0.32-1.31) |
| Years of practice |  |  |  |  |  |  |
| ≤5 | Reference | Reference | Reference | Reference | Reference | Reference |
| 6-10 | 0.80 (0.62-1.03) | 0.77 (0.52-1.15) | 0.83 (0.6-1.16) | 0.71 (0.56-0.90)** | 0.72 (0.49-1.05) | 0.72 (0.54-0.96)* |
| 11-20 | 0.62 (0.47-0.81)*** | 0.62 (0.41-0.94)* | 0.63 (0.44-0.91)* | 0.57 (0.44-0.73)*** | 0.57 (0.39-0.86)** | 0.57 (0.41-0.80)*** |
| ≥21 | 0.70 (0.48-1.04) | 0.67 (0.38-1.17) | 0.79 (0.46-1.35) | 0.57 (0.40-0.82)** | 0.58 (0.33-0.99)* | 0.57 (0.35-0.93)* |
| Leadership role |  |  |  |  |  |  |
| Yes | Reference | Reference | Reference | Reference | Reference | Reference |
| No | 0.51 (0.41-0.63)*** | 0.53 (0.40-0.70)*** | 0.48 (0.34-0.66)*** | 0.50 (0.41-0.60)** | 0.52 (0.4-0.67)*** | 0.50 (0.38-0.66)*** |
| Monthly pay in previous year, RMB |  |  |  |  |  |  |
| <5000 | Reference | Reference | Reference | Reference | Reference | Reference |
| 5000-7999 | 1.12 (0.92-1.37) | 0.96 (0.70-1.30) | 1.26 (0.97-1.65) | 1.15 (0.95-1.39) | 1.12 (0.82-1.52) | 1.17 (0.91-1.50) |
| 8000-11999 | 1.79 (1.42-2.25)*** | 1.6 (1.13-2.27)** | 1.93 (1.42-2.62)*** | 1.47 (1.19-1.83)*** | 1.52 (1.08-2.14)* | 1.45 (1.09-1.92)** |
| ≥12000 | 2.20 (1.63-2.97)*** | 1.56 (1.01-2.41)* | 3.12 (2.04-4.77)*** | 2.08 (1.58-2.74)*** | 2.11 (1.38-3.22)*** | 2.07 (1.43-2.98)*** |
| Working hours per week |  |  |  |  |  |  |
| ≤40 | Reference | Reference | Reference | Reference | Reference | Reference |
| 41-50 | 0.85 (0.62-1.15) | 0.94 (0.62-1.44) | 0.76 (0.49-1.19) | 0.88 (0.67-1.16) | 0.91 (0.61-1.36) | 0.83 (0.57-1.22) |
| 51-60 | 0.78 (0.56-1.08) | 0.84 (0.53-1.35) | 0.70 (0.43-1.14) | 0.71 (0.53-0.96)* | 0.76 (0.49-1.18) | 0.66 (0.44-0.99)* |
| ≥61 | 0.62 (0.46-0.85)** | 0.71 (0.46-1.10) | 0.55 (0.35-0.87)** | 0.58 (0.44-0.77)*** | 0.58 (0.39-0.88)* | 0.56 (0.38-0.83)** |

Notes: * P < .05; ** P < .01; *** P < .001
